# Wnt Pathway in Bone Repair and Regeneration – What Do We Know So Far

**DOI:** 10.3389/fcell.2018.00170

**Published:** 2019-01-07

**Authors:** Khosrow S. Houschyar, Christian Tapking, Mimi R. Borrelli, Daniel Popp, Dominik Duscher, Zeshaan N. Maan, Malcolm P. Chelliah, Jingtao Li, Kamran Harati, Christoph Wallner, Susanne Rein, Dominik Pförringer, Georg Reumuth, Gerrit Grieb, Sylvain Mouraret, Mehran Dadras, Johannes M. Wagner, Jungul Y. Cha, Frank Siemers, Marcus Lehnhardt, Björn Behr

**Affiliations:** ^1^Department of Plastic Surgery, BG University Hospital Bergmannsheil, Ruhr University Bochum, Bochum, Germany; ^2^Department of Surgery, Shriners Hospital for Children-Galveston, University of Texas Medical Branch, Galveston, TX, United States; ^3^Department of Hand, Plastic and Reconstructive Surgery, Burn Trauma Center, BG Trauma Center Ludwigshafen, University of Heidelberg, Heidelberg, Germany; ^4^Division of Plastic and Reconstructive Surgery, Department of Surgery, Stanford School of Medicine, Stanford, CA, United States; ^5^Division of Hand, Plastic and Reconstructive Surgery, Department of Surgery, Medical University of Graz, Graz, Austria; ^6^Department of Plastic Surgery and Hand Surgery, Technical University Munich, Munich, Germany; ^7^State Key Laboratory of Oral Diseases and Department of Oral Maxillofacial Surgery, West China Hospital of Stomatology, Sichuan University, Chengdu, China; ^8^Department of Plastic and Hand Surgery-Burn Center-Clinic St. Georg, Leipzig, Germany; ^9^Clinic and Policlinic of Trauma Surgery, Klinikum Rechts der Isar, Technical University Munich, Munich, Germany; ^10^Department of Plastic and Hand Surgery, Burn Unit, Trauma Center Bergmannstrost Halle, Halle, Germany; ^11^Department of Plastic Surgery and Hand Surgery, Gemeinschaftskrankenhaus Havelhoehe, Teaching Hospital of the Charité Berlin, Berlin, Germany; ^12^Department of Periodontology, Service of Odontology, Rothschild Hospital, AP-HP, Paris 7 – Denis, Diderot University, U.F.R. of Odontology, Paris, France; ^13^Orthodontic Department, College of Dentistry, Yonsei University, Seoul, South Korea

**Keywords:** Wnt, β-catenin, canonical, non-canonical, regeneration, repair, stem cells, bone

## Abstract

Wnt signaling plays a central regulatory role across a remarkably diverse range of functions during embryonic development, including those involved in the formation of bone and cartilage. Wnt signaling continues to play a critical role in adult osteogenic differentiation of mesenchymal stem cells. Disruptions in this highly-conserved and complex system leads to various pathological conditions, including impaired bone healing, autoimmune diseases and malignant degeneration. For reconstructive surgeons, critically sized skeletal defects represent a major challenge. These are frequently associated with significant morbidity in both the recipient and donor sites. The Wnt pathway is an attractive therapeutic target with the potential to directly modulate stem cells responsible for skeletal tissue regeneration and promote bone growth, suggesting that Wnt factors could be used to promote bone healing after trauma. This review summarizes our current understanding of the essential role of the Wnt pathway in bone regeneration and repair.

## Introduction

Unlike most tissues in the human body, bone is capable of spontaneous scarless repair throughout adult life. Skeletal tissue heals following injury by producing new bone with structural geometry and biomechanical integrity (Tarantino et al., 2011) indistinguishable from the surrounding bone (Arvidson et al., 2011). The process of fracture healing in the adult skeleton recapitulates embryonic bone development and is considered a form of tissue regeneration (Ferguson et al., 1999). It is a complicated metabolic process, involving certain regenerative patterns and changes in the expression of 1000s of genes (Marsell and Einhorn, 2011). Disruption to this highly coordinated process can result in delayed or impaired healing (Victoria et al., 2009) leading to mal-union or ‘Non-union.’ Numerous pre-, intra-, and post-operative factors have been found to be associated with impaired bone healing (Panteli et al., 2015), including excessive periosteal stripping, damage to surrounding soft tissue, inadequate post-traumatic or post-operative immobilization, repeated manipulations, and excessive early motion at fracture sites (Victoria et al., 2009). The exact molecular mechanisms of delayed fracture healing, however, are unknown.

Fracture repair is regulated by multiple growth factors (Lieberman et al., 2002). The Wnt signaling pathway has well-known and central roles in bone development, homeostasis, as well as bone repair and regeneration following injury (Xu et al., 2014). Wnt ligands stimulate bone growth, suggesting a strong regulatory role for canonical Wnt signaling pathway in bone healing and highlighting its potential as a therapeutic target to augment fracture healing. A number of molecules able to enhance the canonical Wnt signaling have shown promise in pre-clinical and clinical trials (Secreto et al., 2009).

In this paper we review the canonical Wnt signaling pathway and its role in bone regeneration and repair. A provide an overview of the Wnt pathway and discuss specific canonical Wnt-signaling molecules that may offer favorable targets for facilitating bone repair and regeneration.

## Formation of Bone During Embryological Development

In the early stages of embryonic development, the skeleton is composed of fibrous membrane and hyaline cartilage (Wang M. et al., 2017). By the sixth or seventh week of embryonic life ossification (osteogenesis), begins (Rivas and Shapiro, 2002). Skeletogenesis involves the combined action of numerous genetic programs governing vasculogenesis (Ingber and Levin, 2007), and the specification, proliferation, differentiation, programmed cell death, and remodeling of the ECM. These processes are underpinned by key molecular pivots (Table 1), and as the molecular orchestra responsible for bone formation in the fetus also plays a role in adult skeletal repair (Gadjanski et al., 2012), these pivots represent potential therapeutic targets (Table 2).

**Table 1:** Key molecules and cells involved in bone repair.

Key factors	Function	*In vivo* and *in vitro* effects
**Extracellular messengers**		
IL-1, IL6, TNFα	Elicit inflammation and migration	*In vitro* inhibit osteoblastic differentiation, but *in vivo* TNFα is crucial for bone repair; role of IL-6 is controversial (anti-or pro-osteogenic probably, depending on soluble IL-6 receptor)
TGFβ	Mitogenic factor, osteogenic factor	Can induce osteoblast differentiation at the early stage of immature cells but can also inhibit osteogenesis in committed cells
BMP2	Osteogenic factor	Osteochondrogenic factor; might initiate bone formation and bone healing and can induce expression of other BMPs
BMP4	Osteogenic factor	Osteochondrogenic factor *in vivo* and *in vitro*
BMP7	Osteogenic factor	Osteogenic factor *in vivo* and *in vitro*; active on more mature osteoblasts
SDF1	Chemotactic factor	Allows MSCs homing both *in vitro* and *in vivo*
Noggin	BMP2, 4, and 7 specific inhibitor	Suppresses osteoblastic differentiation
FGFb	Angiogenic and mitogenic factor, osteogenic factor (controversial)	Mutations induce chondrodysplasia and craniosynostosis; can stimulate Sox9; might be a negative regulator of postnatal
IGF-I, II	Mitogenic factors, osteogenic factors	Stimulates growth plate formation, endochondrate ossification and bone formation by osteoblasts
PlGF	Angiogenic and vasculogenic factor	Induces proliferation and osteogenic differentiation of MSCs; crucial for vascularization
VEGF	Angiogenic and vasculogenic factor	Most potent angiogenic and vasculogenic factor; crucial at the onset of bone formation
PDGF	Mitogenic and chemotactic factor	Highly mitogenic factor for MSCs and chemotactic for MSCs, osteoblasts and perivascular cells
Wnts	Mitogenic and osteogenic factors	Depending on Wnt type, crucial for osteoprogenitor proliferation; can also inhibit final osteoblast maturation
DKK1	Inhibitor of Wnt signaling	Strongly inhibits osteogenesis of MSC and osteoprogenitor cells; can stimulate terminal maturation
Ihh	Osteochondrogenic factor	Pivotal role for growth plate and endochondral formation; can inhibit osteoblast differentiation; might induce PTHrP expression
PTHrP	Osteochondrogenic factor	Pivotal role for growth plate and endochondral formation; can induce or inhibit osteogenesis
OPG	Decoy receptor of RANKL, inhibition of RANKL	Strongly inhibits bone resorption and has a pivotal role in bone remodeling
RANKL	Induces osteoclastogenesis	Strongly stimulates bone resorption and has a pivotal role in bone remodeling
M-CSF	Induces osteoclastogenesis	Crucial for osteoclastogenesis
Gastrointestinal serotonin	Neurotransmitter inhibiting osteogenesis	Expressed by enterochromatin cells, inhibits bone formation and repressed by Lrp5
**Intracellular messengers**		
PKA/CREB	Transduce osteogenic signaling	Can transduce osteogenic signaling (still controversial); possible indirect effect
MAPKs	Transduce osteogenic signaling by phosphorylation	Crucial for regulation of intracellular signaling induced by osteogenic factors (still controversial)
β-Catenin	Osteogenic transducer factor	Pivotal role in transducing osteogenic signal from Wnt and is negatively regulated by GSK3β
Runx2	Early osteogenic transcription factor	Master regulator of early osteogenesis; runx2 mice died, with no bone formation
Osterix	Late osteogenic transcription factor	Master regulator of late osteogenesis, inhibiting chondrogenesis
Dlx5	Osteogenic homeobox protein	Induces osteoblast maturation but inhibits osteocyte formation
Msx2	Osteogenic homeobox protein	Induces proliferation of immature cells; responses depend on Dlx5 quantity
NF-kB	Inflammation transducer factor, inhibits osteogenesis	Inhibits the differentiation of MSCs and committed osteoblastic cells
**Cells**		
MSCs	Origin of osteoblasts	Can form bone *in vivo* and osteoblasts *in vitro*
Osteoblasts	Osteogenic professional cells	Generate bone formation
Adipose tissue-derived stromal cells	Multipotential cells	Can give rise to bone *in vivo* and *in vitro* but are less effective than bone marrow MSCsl


**Table 2:** Clinical relevance of key factors in bone repair.

Key factors tested	Observations
BMP2	Used for spine fusion, bone non-union and bone defects; clinically efficient for bone repair and regeneration; some adverse effects observed (osteolysis and ectopic bone formation)
BMP7	Used for spine fusion and bone non-union; clinically efficient for bone repair
PTHrP/PTH	Used for osteoporosis; efficient for increasing bone mass when intermittently administered
Wnt-β-catenin	LiCl used as a specific inhibitor of GSK3β to increase bone mass post-fracture and to diminish fracture risk Bortezomib, proteasome inhibitor used in treatment of multiple myeloma (MM); also increases bone mass Anti-DKK1 monoclonal antibody (BHQ880) used to inhibit osteolysis in MM or to increase BMD Anti-sclerostin antibody used to increase bone mass
RANKL/OPG	Targeting RANKL to treat osteoporosis; e.g., denosumab (anti-RANKL antibody), which can be used with biphosphonates
Biphosphonates	Widely used for osteoporosis, bone necrosis, osteogenesis imperfecta and some osteolytic tumors (MM) (zoledronate, alendronate, risedronate); some adverse effects noted (osteonecrosis, inhibition of osteogenesis)
TGFβ	Used as a bone non-union marker
Platelet-rich plasma	Used in maxillofacial surgery and for bone defects with or without biomaterials with or without osteoregenerative cells
MSCs or osteoblasts	*In vitro*-expanded MSCs (or osteoblasts) used for bone defects, osteonecrosis, immune rejection; randomized controlled clinical trials are required


**FIGURE 1:**
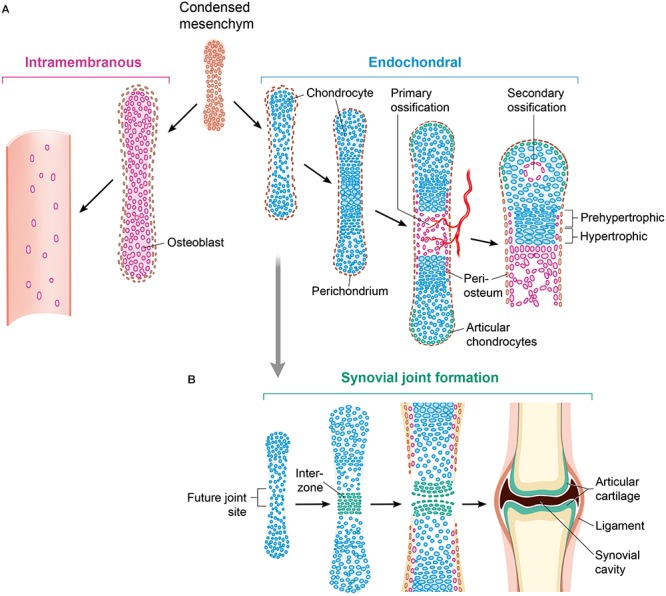
Ways of bone formation. **(A)** Ossification can occur via endochondral or intramembranous mechanisms. As part of the intramembranous ossification, mesenchymal cells differentiate directly into osteoblasts and generate bone tissue. Chondrocytes develop from mesenchymal cell differentiation with forming an intermediate cartilage during endochondral ossification. By mineralizing the matrix, undergoing apoptosis and attracting blood vessels and osteoblasts, hypertrophying chondrocytes that stop proliferating initiate a centric growth plate. **(B)** Histologically detectable flattening and gathering of cells that are forming an interzone, is the first sign of joint formation. This is followed by maturation and remodeling leading to a mature synovial joint. The Wnt signaling pathway is crucial for controlling almost all aspects of this skeleton formation. Osteoblasts (purple); chondrocytes (blue); osteochondroprogenitor cells (brown).

Together, bone and cartilage comprise skeleton, and are produced by osteoblasts and chondrocytes, respectively (Regard et al., 2012). During embryological development, bone is formed by (1) intramembranous and (2) endochondral ossification (Figure 1A). During embryonic development skeletal elements are separate in places to form joints, critical structures for mobility. Synovial allow for movement between boney fronts, and form upon the dedifferentiation and flattening of chondrogenic cells in newly formed cartilage, which creates an interzone (Figure 1B).

## Mechanisms of Bone Repair and Regeneration

The main function of the skeleton is structural; it creates a strong, protective, mechanically optimal structure for more delicate organs and soft tissues (Oryan et al., 2015). Bone tissue constantly adapts to biomechanical loading and environmental stress (Ozcivici et al., 2010) through two opposing but synergistic processes; bone resorption and bone formation (Feng and McDonald, 2011).

Bone repair following damage is a complex and well-organized regenerative process initiated in response to injury which effectively restores skeletal function (Morgan et al., 2014). Unlike other adult tissues, which generate scar tissue in response to injury, the skeleton undergoes regenerative healing, forming new bone indistinguishable from adjacent, uninjured tissue (Colnot et al., 2003). Fracture healing mimics early developmental processes and occurs by both direct and indirect repair (Secreto et al., 2009). Direct (primary) repair is possibly when the bony fronts of adjacent bones are in close contact. This is usually the case after surgical treatment with stable fixation of the injury (Pesce et al., 2009). Osteoprogenitor cells, osteoclasts, and undifferentiated mesenchymal stem/stromal cells (MSCs) recruited to the fracture site may also promote bone formation in a mechanism similar to formation of bone during intramembranous ossification in the skull and clavicles (Wu et al., 2016). During indirect (secondary) healing, bone formation is akin to endochondral ossification, the developmental method by which long bones are originally made in development (Long and Ornitz, 2013). Following injury, a soft callus forms composed of largely inflammatory cells. This callus develops into an intermediate cartilaginous template which subsequently undergoes calcification, and ultimately is replaced by woven bone (Marsell and Einhorn, 2011) and then lamellar bone through a remodeling process that takes several months before. The resulting lamellar bone is able to support normal load bearing (Marsell and Einhorn, 2011). With surgical fixation, temporary immobilization, or both, most fractures heal after several months. However, three to 10% of fractures fail to heal and result in the formation of a fibrous or non-union (Kloen et al., 2012). The rate of successful fracture healing may be increased, and the time of healing decreased, by therapies that induce bone formation at the break point (Hoang-Kim et al., 2009).

### Three Wnt Signaling Pathways

Wnt signaling is a pathway that has been conserved over evolution. It regulates important aspects of cell polarity, cell fate determination, cell migration, formation of the primary axis, organogenesis, and the renewal of stem cells during embryonic development (Komiya and Habas, 2008). Dysregulation of Wnt signaling has been implicated in many diseases, including autoimmune diseases and cancer (Shi et al., 2016).

The name Wnt originates from the fusion of *wingless*, the segment polarity gene of the *Drosophila*, and *integrated* (int-1), the vertebrate homolog (van Amerongen and Nusse, 2009). Wnt ligands, which are encoded by 19 Wnt genes, are cysteine rich highly hydrophobic proteins, 320–400 amino acid base pair in length, with an N-terminal signal peptide for secretion, and a high degree of sequence homology (Wang et al., 2018). The Wnt ligands bind receptors on the cell surface of recipient cells to activate the Wnt pathway by triggering intracellular signaling cascades which orchestrate numerous cell biological and developmental processes (Willert and Nusse, 2012), important in many physiological settings (MacDonald et al., 2009). Due to thier hydrophobic natuextrre, Wnt proteins are found in association with cell membranes and the ECM. They become palmitoylated in the endoplasmic reticulum of Wnt-producing cells in the presence of acyltransferase porcupine (Herr and Basler, 2012). This palmitate modification is thought to assist in ligand reception on Wnt-responding cells (Mikels and Nusse, 2006). Modified Wnt proteins are then transported and secreted in secretory vesicles which are under control by Wntless/Evi (evenness interrupted) – the multi-pass transmembrane protein present in the plasma membrane and/or the Golgi apparatus (Ching and Nusse, 2006). This facilitates the release of Wnt protein from the cells and thus their association with the seven-pass transmembrane receptor Frizzled (Fzd) (Maupin et al., 2013). Fzd is present on the surface of responding cells and possesses a large extracellular domain, the ‘cysteine-rich domain’ – made of 10 cysteine residues in a conserved motif’(Huang and Klein, 2004). Low-density lipoprotein receptor-related proteins 5 or 6 (LRP5/6) or ROR act as co-receptors to Fzd and assist the binding between Wnt proteins and the receptor (MacDonald and He, 2012). The co-receptor engaged then determines the downstream effect of the successful ligand binding, initiating either the non-canonical or the canonical pathways (Mohammed et al., 2016). As the Wnt signaling pathway is fundamental during embryological development, the expression of Wnt proteins and antagonists happens under strict temporal and spatial regulation (Komiya and Habas, 2008).

**FIGURE 2:**
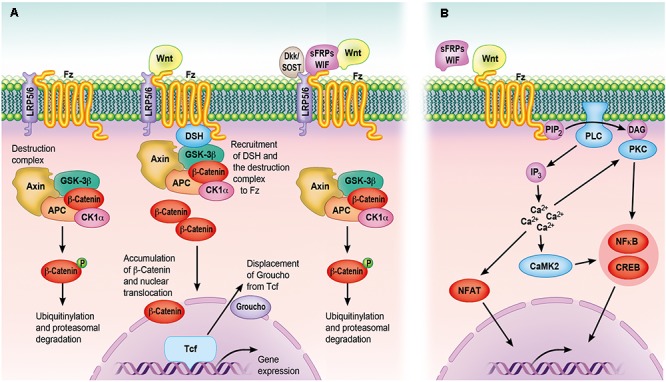
The Wnt signaling cascades. **(A)** The canonical Wnt signaling cascade depends on β-catenin, which serves as an intracellular signaling molecule. In case Wnt is not binding to Fz receptors, β-catenin is sequestered into a destruction complex composed of Axin, CK1α, APC and GSK3β, phosphorylated, ubiquitinylated and subsequently degraded by the proteasome. Following the binding of Wnt to Fz receptors and LRP5/6 co-receptors, DSH recruits the destruction complex to the cell membrane by interacting with the receptor complex. This allows newly synthesized β-catenin to accumulate within the cytoplasm and to translocate to the nucleus. By displacing the transcriptional co-repressor groucho from TCF transcription factors, nuclear β-catenin can activate a gene transcription program, whereas Wnt-binding antagonists (sFRPs/WIF) and Wnt receptor antagonists (Dkk/SOST) inhibit the canonical cascade. **(B)** The non-canonical Wnt signaling cascade is characterized by the activation through phosphorylation cascades, which are themselves activated by specific ligand–receptor interactions, seemingly without engagement of the LRP co-receptors. Increasing intracellular Ca^2+^ levels following PLC- and DAG production can trigger many of these cascades. Subsequently, PKC and CaMKII can activate transcription factors like NFκB and CREB, mediated IP3 and calmodulin is involved in the activation of NFAT. However, only the Wnt-binding antagonists are able to inhibit the non-canonical cascade. APC, adenomatous polyposis coli; CaMKII, calcium/calmodulin-dependent protein kinase type II; CK1α, caseine kinase 1-α; CREB, cyclic AMP-responsive element-binding protein; DAG, diacylglycerol; Dkk, Dickkopf; DSH, disheveled; GSK3β, glycogen synthase kinase-3 β; IP3, inositol 1,4,5-triphosphate; LRP, low-density lipoprotein receptor-related protein; NFAT, nuclear factor of activated T cells; NFκB, nuclear factor κB; PIP2, phosphatidylinositol 4,5-bisphosphate; PKC, protein kinase C; PLC, phospholipase C; sFRPs, secreted frizzled-related proteins; SOST, sclerostin; WIF, Wnt inhibitory factor.

Intracellular Wnt signaling is categorized into least three main pathways: (1) the β-catenin dependent pathway (also called the ‘canonical Wnt pathway’); (2) the planar cell polarity (PCP) pathway; and 3. the Wnt/Ca^2+^ pathway (Houschyar et al., 2015). In the canonical Wnt signaling pathway, the ubiquitination and degradation of β-catenin mediated by glycogen synthase kinase 3 (GSK-3) is inhibited (Gao et al., 2014). In the PCP pathway, Wnt signaling activates jun N-terminal kinase (JNK) and this results in cytoskeletal rearrangements into an asymmetrical organization, as well as polarization of cell morphology within the plane of epithelial sheets (Geetha-Loganathan et al., 2008). This pathway shares many components of the canonical Wnt pathway including Frizzled, and the downstream components GTPase Rho and a kinase cascade including Misshapen, JNK kinase, and JNK (Habas and Dawid, 2005). GSK-3 and adenomatous polymosis coli (APC) of the canonical Wnt signaling pathway are also involved in spindle orientation and asymmetric cell division of *C. elegans* and *Drosophila* (Wu and Herman, 2006). In the Wnt/Ca^2+^ pathway, Wnt is involved in the release of intracellular calcium, possibly via G proteins (Lu and Carson, 2009; Thrasivoulou et al., 2013). This pathway includes activation of Phospholipase C (PLC), protein kinase C (PKC), and calmodulin-dependent kinase II, and has a role in *Xenopus* ventralization and in the regulation of convergent extension movements (Kestler and Kuhl, 2008). The canonical Wnt signaling pathway is the best characterized and is strongly implicated in skeletal tissue regeneration and repair (Clevers, 2006) (Figure 2).

## Canonical Wnt Signaling Pathway

Recent investigation into the canonical Wnt pathway has led to novel insights into the various levels of canonical Wnt signaling whichhave refined the model of how this pathway is regulated (Zhan et al., 2017). At least seven of 19 Wnt proteins (Wnts 1, 2, 3a, 3b, 4, 8, and 10b), can activate this pathway (Chen et al., 2015). Cannonical Wnt signaling results in the accumulation and translocation of Beta-catenin (β-catenin), into the nucleus (Enzo et al., 2015). β-catenin is an adherens junction-associated protein and functions to: (1) enable cell–cell adhesion; and (2) mediate intracellular Wnt signaling (Valenta et al., 2012). Intranuclear accumulation of β-catenin activates transcription factors that target specific genes that mediate cellular development (Cadigan and Waterman, 2012). Dysregulation of β-catenin signaling is implicated in a number of malignancies, suggesting its important role in the control of cellular proliferation and/or cell death (Tarapore et al., 2012). In the absence of Wnt ligands, cytoplasmic β-catenin is degraded by a multiprotein complex made of Axin, casein kinase 1 (CK1), APC and GSK3 (Stamos and Weis, 2013). CK1 and GSK3 phosphorylate β-catenin in the NH2-terminal degradation box, targeting it for ubiquitination (Stamos and Weis, 2013). bTRCP1 (a component of ubiquitin E3 ligase) or bTRCP2 complex the ubiquinate phosphorylated β-catenin for proteasome-mediated degradation by the β-catenin destruction complex (Reischl et al., 2007).

The canonical Wnt pathway is activated by binding of specific Wnt ligands to the Fzs along with the LRP-5/6 co-receptors (Gao et al., 2014). However, Wnt intracellular signaling is complex (Sethi and Vidal-Puig, 2010); there are 10 known human Fz receptors to date (Shevtsov et al., 2006), and although the role of Fz in acting as a receptor for Wnts has long been known, the role of LRP-5 and its homolog LRP-6, acting as co-receptors for Wnt proteins has only recently been established (MacDonald and He, 2012). The Dickkopf (Dkk) family are secreted proteins which bind LRP-5 or LRP-6 with high affinity can therefore directly antagonize canonical Wnt binding (MacDonald and He, 2012). Upon the successful binding of Wnt with its receptors, the intracellular protein, Dvl, is activated. Dvl transduces the membrane signal from the receptor complex (Gonzalez-Sancho et al., 2004) by inhibiting GSK-3b, leading to the collapse of the multi-protein β-catenin destruction complex (Medina and Wandosell, 2011). Consequently, β-catenin is not phosphorylated and targeted for proteasome mediated degradation and is able to accumulate in the cytoplasm and translocate to the nucleus. Intranuclear β-catenin then associates with members of the T cell factor/lymphoid enhancer factor (TCF/LEF) family and together they activate the transcription of numerous genes involved in a range of functions, for example c-myc and cyclin D1 (Ma and Hottiger, 2016).

The first indication of a link between bone biology and canonical Wnt signaling was discovered more than one decade ago (Baron and Kneissel, 2013). Mutations in the Wnt signaling cascade were found to result in excessive bone growth or in excessive resorption (Yavropoulou and Yovos, 2007): loss of function mutations of the co-receptor LRP5 causes syndromes characterized by low bone mass and consequently frequent bone fractures (Pinzone et al., 2009); alternatively, the gain of function mutations of LRP5 receptor lead to high bone mass (Balemans and Van Hul, 2007). These findings are further corroborated by the association of SNPs of the LRP5 gene with reduced bone mineral density (BMD) and an elevated risk of osteoporotic fractures (Schulze et al., 2010). LRP5 and LRP6 also transduce Wnt signaling *in vitro* and indicated overlapping roles during *in vivo* skeletal patterning (Cui et al., 2011). Although LRP5/6 regulate bone mass, the mechanism by which they do so is yet to be fully elucidated.

Recent research shows that that gene variation in Wnt-16 has also been linked with decreased BMD and osteoporotic fractures; Wnt-16 knockout mice have a substantial decrease in bone thickness (Zheng et al., 2012). The initial phase of skeletal tissue repair or active bone remodeling is similar to that occurring during skeletal embryogenesis as skeletal stem cells are shuttled to either the osteogenic or the chondrogenic route (Bianco and Robey, 2015). One study reporting on the Wnt involvement in fracture repair identified upregulation of Wnt5A, β-catenin, FZD, and numerous target genes following injury (Komatsu et al., 2010). A later follow-up study demonstrated upregulation of additional Wnt related markers such as Wnt5B, LRP5, Disheveled (Dvl), TCF1 and peroxisome proliferator-activated receptor delta (PPARD) (Tamura et al., 2010). In contrast, the transcription factor LEF1 was repressed during the initial phases of bone repair, and the stage at which maximal bone was formed (Shahi et al., 2017). However, LEF1 inhibits RUNX2-dependent activation of OCN in osteoblasts. RUNX2 is the transcription factor needed for development of the osteoblast. This suggests that decreased LEF1 expression is necessary for bone repair to occur (Rahman et al., 2015). As described above, β-catenin has various roles at different stages of bone repair. In the early phases following injury, β-catenin regulates the ratio of osteoblasts and chondrocytes present in the callus which arises from pluripotent MSCs (Bao et al., 2017). Later in the bone healing process, β-catenin induces differentiation of osteoblasts and osteoblastic matrix production (Wang T. et al., 2017). LRP5 and β-catenin gene expression is upregulated in cells present in the fracture callus. β-catenin is also expressed in proliferating periosteal osteoprogenitor cells, chondrocytes, as well as osteoblasts, which suggests the canonical Wnt signaling pathway is active in both endochondral and intramembranous ossification (Komatsu et al., 2010; Lin and Hankenson, 2011; Regard et al., 2012). Recent work has corroborated this hypothesis; fractured long bones of LRP5 knockout mice are reduced in size, have decreased BMD, and are biomechanically inferior to the long bones of wild-type (WT) littermates (Komatsu et al., 2010). Furthermore, administration of the Wnt antagonist, DKK1 antibody increased the size of the fractured tissue, as well as its BMD and biomechanical properties. This illustrates how ablation of the Wnt-LRP5 interaction delays the reestablishment of biomechanical integrity during bone repair, and that the canonical Wnt pathway, and specifically the LRP5 coreceptor, are key components of fracture repair.

The non-canonical Wnt pathways also contribute to intramembraneous and endochondral ossification following fracture (Heilmann et al., 2013). Wnt-5a is a non-canonical Wnt ligand and has been found to play an integral role in BMP2-mediated osteogenic differentiation (Nemoto et al., 2012). During osteogenic differentiation, BMPs act to downregulate Wnt signaling via sclerostin and Dkk-1 (Kamiya et al., 2008; Zhang et al., 2016b). Absence of the BMP receptor type 1 in osteoblasts of mice results in decreased levels sclerostin and Dkk-1 and increased bone mass (Kamiya et al., 2008). The Wnt-antagonizing effects of BMP led to the suggestion that Smad1 forms a complex with, and thus sequesters, Dvl (Liu et al., 2006). However, understanding the balanced interplay between the BMPs and Wnt ligands are still under intense investigation.

Activation of the Notch pathway inhibits Wnt/β-catenin-induced osteogenic differentiation (Cao et al., 2017). Overexpression of the Notch intracellular domain, both *in vivo* and *in vitro*, is associated with reduced Wnt signaling and impaired osteoblastogenesi (Lin and Hankenson, 2011). The Hedgehog (Hh) works upstream of the Wnt pathway sequentially and promote the osteogenic differentiation of MSCs (James, 2013), and is thus proposed to regulate the early stages of osteogenic differentiation of MSCs (Beederman et al., 2013). Inhibition of Wnt signaling reduces Hh-induced osteogenic activity in both *in vitro* and *in vivo* models (Huang et al., 2007).

Wnt signaling is also involved in osteoimmunomodulatory pathways. Of note, tumor necrosis factor (TNF)-α promotes the activity Dkk-1 and thus block osteoblast differentiation (Diarra et al., 2007). Mice overexpressing TNFα have a rheumatoid arthritis-like destruction of their joints (Baum and Gravallese, 2014). Antibody mediated Dkk-1 neutralization in the TNFα transgenic mice rescues the joint destruction and even results in the formation of osteophytes (Diarra et al., 2007). The balance between skeletal bone formation and resorption and the interaction between the Wnt pathway and TNFα-induced inflammatory process, is complex.

There is increasing evidence of crosstalk between the Wnt pathway and other signaling pathways. For example, Wnt pathways reciprocally regulate the progranulin growth factor in frontotemporal dementia (Rosen et al., 2011). Progranulin, or ‘proepithelin,’ is a newly identified growth factors able to promote the differentiation of MSCs into chondrocytes as well as endochondral ossification (Wu et al., 2011). The interplay between Wnts and progranulin in osteogenesis are a subject of future investigations.

Wnt signaling found to induce osteogenic differentiation via changing MicroRNA (miRNA) (Kureel et al., 2018). A number of different miRNA molecules can promote or inhibit MSC mediated osteogenic differentiation (Kang and Hata, 2015). miRNA function to interact with several growth factors and transcriptional factors such as Runx2 and osterix, at various stages of osteogenic differentiation (Vimalraj and Selvamurugan, 2013). Several miRNAs specifically interact with Wnt ligands, with a consequent effect on osteogenesis (Peng et al., 2016); miR-27 inhibits APC, and thus canonical Wnt signaling and promotes bone formation (Wang and Xu, 2010), and miR-335-5p downregulates Dkk-1 and thus promotes osteogenic differentiation (Zhang et al., 2017).

## Mesenchymal Stem/Stromal Cells (MSCs) and Wnt Signaling in Bone Development and Homeostasis

Mesenchymal stem/stromal cells are multipotent progenitor cells with that ability to into multiple tissue types, including bone, cartilage, fat, tendon, and muscle (Klimczak and Kozlowska, 2016). MSC fate and self-renewing potential, transient amplifying activity is under the influence of the MSC microenvironment and systemic factors (Crane and Cao, 2014). MSCs populate various anatomical locations including the bone marrow and fat, and their impressive differentiation capacity makes them a favorable therapeutic option (Chanda et al., 2010). The ability to promote osteogenic differentiation of MSCs, either prior or post-transplantation, may serve as an effective therapy to promote bone formation in areas of deficiency (Wagner et al., 2011). In the 1960s and 1970s, Friedenstein et al. (1970) were first to describe the rare population (∼0.0001%) of nucleated cells in the bone marrow which adhere to plastic, form cells of spindle-shaped morphology, and rise to round-shaped fibroblastoid colonies (colony-forming unit-fibroblasts or ‘CFU-Fs’). Freidenstein (1990) also demonstrated that the bone marrow derived cells have the capacity to differentiate into bone, cartilage, and/or adipose tissue upon *in vivo* transplantation.

The commitment of MSCs down a certain cell lineage is under the control of a collection of growth factors, but current understanding of the processes influencing cell fates is limited (Li et al., 2011). Studies in both mice and humans show that MSCs can augment bone regeneration by differentiating into osteoblasts as well as by secreting osteogenic growth factors and anti-inflammatory cytokines (Zwingenberger et al., 2013). Granero-Moltó et al. (2009) transduced MSCs to express firefly luciferase and show that MSCs migrate toward the fracture site via the CXCR4 receptor and then promote healing by increasing the cartilage and bone content of the callus, thus altering its biomechanical properties. A clinical study reported on the bone-healing effects of MSCs when used as treatment of defects of long bones, with beneficial effects still evident 7-years later (Gjerde et al., 2018). Another clinical study demonstrated the beneficial effects on injecting MSCs along with bisphosphonates to treat femoral head core decompression and avascular necrosis (Gianakos et al., 2016). Injection of an antagonists against the chemokine CC receptor (CCR1) reveals that this receptors is an important chemoreceptor directing MSC migration and osteoblastic differentiation (Gibon et al., 2012). Osteoporosis is a systemic bone disease largely affecting the elderly population. Glucocorticoid-induced osteoporosis in rats can be prevented through systemic administration of allogenic MSCs via their osteoblastogenic effects. Together these data suggest MSCs undergo osteoblastic differentiation and promote a more regenerative inflammatory state, and this may have therapeutic implications for a number of diseases of the bone (Pajarinen et al., 2017).

**FIGURE 3:**
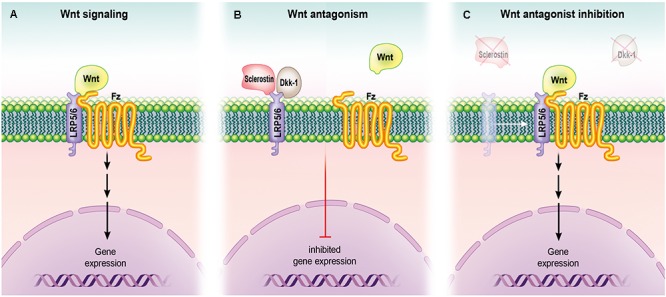
Role of Wnt signaling in osteoblasts. **(A)** Upon binding to its receptor (Frizzled) and co-receptors (LRP5 and LRP6), Wnt activates their signaling pathway, leading to gene expression (and ultimately protein synthesis and the formation of bone). **(B)** Wnt antagonists sclerostin and Dkk-1 bind LRP5 and LRP6, preventing their interaction with Frizzled and resulting in inhibition of gene expression. **(C)** Loss-of-function mutation in a gene that encodes for a Wnt antagonist orpharmacological engagement of the antagonist with an inhibitory molecule such as an antibody can lead to inhibition of Wnt antagonism and promote gene expression.

Wnt signaling pathway has a well-established critical role in promoting osteogenic differentiation of MSCs (Liang et al., 2016). Additionally, Wnt ligands stimulate osteoblast proliferation and support osteoblast maturation (Figure 3). The Wnt signaling pathway is involved in both intramembranous and endochondral ossification (Zhong et al., 2014). Minear et al. (2010) used a mouse model to demonstrate that enhanced Wnt signaling through the delivery of liposomal vesicles containing purified Wnt-3a protein resulted in accelerated fracture healing due to increased proliferation and earlier differentiation of skeletal stem cells/progenitor cells. This highlights the therapeutic potential of using a biochemical strategy through which proteins can be used to deliver Wnt ligands, and thus to increase the duration and strength of the bone healing effect of Wnt signaling. Previously it has been shown that β-catenin can promote the progression of MSCs from osteoblastic precursor cells into more mature osteoblasts and can also suppress the differentiation of MSCs into adipogenic and chondrogenic lineages (Case and Rubin, 2010; Ullah et al., 2015). The canonical Wnt pathway is especially influential in inhibiting the expression of the major adipogenic inducers, PPARγ and CCAAT/enhancer binding protein α, to suppress adipogenic differentiation while upregulating the osteogenic regulators Runx2, Dlx5, and Osterix (Kang et al., 2007). In addition, non-canonical Wnt signaling also induces osteogenic differentiation through a different mechanism (Arnsdorf et al., 2009). The non-canonical ligand Wnt-5a suppresses PPARγ (Topol et al., 2003) and thus inactivates chromatins. Although the interplay between these two independent mechanisms induced by Wnt ligands is still not totally understood, it is evident that Wnt signaling regulates the osteogenic differentiation of MSCs (Zhang et al., 2013).

Bone morphogenic proteins (BMPs), mainly BMPs 2, 6, and 9, are potent growth factors which stimulate MSCs to undergo differentiation into osteocytes (Scarfi, 2016). There is substantial crosstalk between BMP and Wnt signaling (Lin and Hankenson, 2011); different BMPs either enhance or antagonize Wnt-induced osteogenic differentiation (Itasaki and Hoppler, 2016; Wu et al., 2016), BMP-induced osteogenic differentiation of MSCs is dependent upon functional Wnt signaling (Tang et al., 2009), and the Wnt and BMP pathways share common targets, such as the connective growth tissue factor (Luo et al., 2004; Si et al., 2006). The osteogenic effects of BMP9 are enhanced by Wnt-3a and inhibited by β-catenin knockdown or overexpression of FrzB, which is a Fzd antagonist (Boland et al., 2004). The ability of BMP2 to induce ectopic bone formation is antagonized by Dkk-1 overexpression or conditional knockout of β-catenin (Chen et al., 2007). BMP2 is thought to promote osteogenic differentiation by increasing the expression of LRP5 and stabilizing β-catenin through the downregulation of β-Trcp (Zhang et al., 2009).

Overall, the Wnt and additional signaling pathways interact in an extensive network during osteogenic differentiation regulated by a variety of molecules. Full characterization of all these interactions is yet to be completed. Nevertheless, a better understanding of the intricate trans-pathway crosstalk in osteogenesis is a necessity in order to develop new therapies able to act on these signaling pathways for clinical benefit.

## Opportunities for Therapeutic Use

The ability to control the self-renewal, proliferation, and differentiation of skeletal stem cells could lead to the possibility of expanding a small population of adult progenitor cells and inducing their differentiation in a time sensitive manner to replenish the function of skeletal and cartilaginous tissue (Kodaka et al., 2017). Bone regeneration for fracture repair and defect healing has been a focus of orthopedic surgery (Arvidson et al., 2011). Internal and external fixation at orthotopic sites is the standard of care and achieves short-term stabilization, however, successful long-term stability still requires bone fusion or bone augmentation (Geisler, 2013). Autogenous bone grafting is a common technique to repair large-sized skeletal defects (Oryan et al., 2014), but donor bone is limited in supply and harvesting can cause significant morbidity at the donor site. Additionally transplanted grafts are at risk of infection and failure. Allograft bone may be antigenic and comes with the risk of transmitting disease (Ishikawa et al., 2010), and biomaterials increase the rate of infection and often have suboptimal biomechanical properties (Amini et al., 2012). A cost-effective pharmacologic agent that can be delivered non-invasively is the ideal therapeutic way to promote bone repair and regeneration (Zhang et al., 2016a). Factors BMP-7 (or ‘osteogenic protein 1,’ OP-1) and BMP-2 have been used with increasing success in preclinical and clinical trials (Roberts and Rosenbaum, 2012). Supplementation with these BMPs enhances bone formation, however, they are effective only in excessive quantities and have short half-lives and thus short-term bioavailability. Additionally, there are currently no methods currently able to deliver these proteins allowing their sustained release, and this has hindered progress to the use of BMPs in humans (Chen, 2001).

The Wnt pathway is well-characterized and is thus an attractive therapeutic for bone repair and skeletal homeostasis (Leucht and Helms, 2015; Gomes et al., 2017). Additionally, a substantial body of literature has accumulated supporting the role of Wnt signaling in skeletogenesis and the regulatory functions of Wnt signaling on stem and skeletal cells (Li et al., 2015). Animal models of osteoarthritis have implicated Wnt/β-catenin signaling abnormalities in the changes observed in the cartilage and the bone, and this suggests that the β-catenin pathway may be a therapeutic target for osteoarthritis (Kim et al., 2013). Sclerostin, a SOST gene product expressed by articular chondrocytes and osteocytes, and inhibits Wnt signaling (Lewiecki, 2014). Sclerosteosis and van Buchem disease are rare genetic disorders with low levels of sclerostin and high BMD. Research in animals suggests that sclerostin may be a potential target for the treatment of conditions of characterized by low BMD and increased risk of fractures, such as osteoporosis (Krishnan et al., 2006; Pietrzyk et al., 2017), and sclerostin is being investigated as a treatment for post-menopausal osteoporosis (Lewiecki, 2011). Production of highly specific antibodies to inhibit a ligand or receptor may help to develop effective therapies that are affordable and can thus become widely used products Humanized sclerostin monoclonal antibodies currently being developed include Romosozumab (AMG 785, CDP-7851; co-developed by Amgen, Thousand Oaks, CA, United States, and UCB, Belgium) and Blosozumab (Eli Lilly and Company, Indianapolis, IN, United States). BPS804 (Novartis, Basel, Switzerland), an antisclerostin agent. The interactions between Wnt receptors and co-receptors also represent reasonable therapeutic targets (MacDonald and He, 2012). Dual inhibition of Wnt via the antagonist DKK-1 in animals treated with sclerostin antibody, results in synergistic bone formation in rodents and non-human primates, suggesting that a negative feedback mechanism limits Wnt-driven bone formation (Florio et al., 2016).

Although there are multiple potential benefits of manipulating the Wnt signaling cascade, these should be performed with caution. The Wnt signaling cascade regulates numerous pathological processes, including the development of cancer (Van Camp et al., 2014). The transportation of Wnt proteins to the target is still challenging, since they are hydrophobic and therefore insoluble in aqueous substances. However, Wnt has successfully been purified and packaged into liposomes, circumventing this delivery challenge (Minear et al., 2010). Incubation of L-Wnt3a can further enhance the survival, proliferation, and engraftment efficiency of bone marrow cells, partly by blocking caspase-dependent programmed cell death (Dhamdhere et al., 2014). Besides, other molecules intervening on different components of the canonical Wnt signaling pathway may offer therapeutic potential (Wagner et al., 2011; Zimmerman et al., 2012). One of these is Lithium, which inhibits GSK3 and can thereby increase β-catenin, with promising effects on bone healing (Freland and Beaulieu, 2012). Further investigation may reveal additional molecules able to potentiate the bone-healing effects of the Wnt signaling pathway.

## Conclusion and Future Directions

Wnt signaling during bone regeneration and repair involves a well-organized interaction among various cells and regulatory factors. The ability of adult bone to scarlessly regenerate can be impaired resulting in pathological fractures that become fibrous or fail to unite. The therapeutics developed to promote bone regeneration have focused on stimulating MSCs and their osteogenic differentiation. It is increasingly apparent that Wnt signaling plays a fundamental role during the embryological development of bone and cartilage and, in the adult skeleton, regulates bone homeostasis, repair, and regeneration. The Wnt pathways influence stem cell proliferation, differentiation, and maintenance. Mutations in Wnt genes, receptors, and inhibitors can have detrimental effects on bone formation and turnover, and can result in skeletal abnormalities. Recent progress in understanding the critical roles of Wnt/β-catenin signaling in the development and maturation of skeletal cells has invited opportunities to develop pharmaceutical agents to treat non-unions and accelerate fracture repair. Despite the rapid and measurable accomplishments, the role of the Wnts and Wnt antagonists on skeletal physiology and regeneration remain to be fully elucidated. Clinical trials are currently being undertaken to explore the effects of therapeutic agents manipulating the Wnt signaling pathway on a number of endocrine and orthopedic conditions.

## Author Contributions

KSH conceived of the article and the authors KSH, CT, MRB, DP, DD, ZNM, MPC, JL, KH, CW, SR, DP, GR, GG, SM, MD, JMW, JYC, FS, ML, and BB made equal contributions to its written content.

## Conflict of Interest Statement

The authors declare that the research was conducted in the absence of any commercial or financial relationships that could be construed as a potential conflict of interest.
